# Analyzing Spinal Shape Changes During Posture Training Using a Wearable Device

**DOI:** 10.3390/s19163625

**Published:** 2019-08-20

**Authors:** Katharina Stollenwerk, Jonas Müller, André Hinkenjann, Björn Krüger

**Affiliations:** 1 Hochschule Bonn-Rhein Sieg, Institute of Visual Computing, 53757 Sankt Augustin, Germany; 2 Gokhale Method Institute, Stanford, CA 94305, USA

**Keywords:** posture analysis, spinal posture, accelerometer, wearable sensor

## Abstract

Lower back pain is one of the most prevalent diseases in Western societies. A large percentage of European and American populations suffer from back pain at some point in their lives. One successful approach to address lower back pain is postural training, which can be supported by wearable devices, providing real-time feedback about the user’s posture. In this work, we analyze the changes in posture induced by postural training. To this end, we compare snapshots before and after training, as measured by the Gokhale *SpineTracker*™. Considering pairs of before and after snapshots in different positions (standing, sitting, and bending), we introduce a feature space, that allows for unsupervised clustering. We show that resulting clusters represent certain groups of postural changes, which are meaningful to professional posture trainers.

## 1. Introduction

Back pain is experienced by a large percentage of the world’s population. Approximately 70% of the world’s population experience lower back pain, contributing to the worldwide burden of disease. Levels of intensity and disability vary [1]:

- Grade I: low-intensity, low disability symptoms are experienced by 49%.
- Grade II: high-intensity, low disability symptoms are experienced by 12%.
- Grade III/IV: high-intensity, high disability symptoms are experienced by 11%.

In the U.S., 28% of the American workforce experiences lower back pain of various intensities at any given time, and during any given year 8% of the working population will be disabled due to low back pain [2,3]. According to Rubin and Devon [4], a majority of the population will suffer from a back problem at some point in their lives. This makes back pain the largest factor in the decline in productivity of workers, resulting in estimated economic costs ranging from $200 billion to $600 billion per year in the United States [5].

There is a wide range of treatments for back pain in use. This includes invasive methods, medication, exercise, supportive clothing, and postural change. The crowd-sourcing platform healthoutcome.org collects patients’ ratings about all available treatments. In total more then 160,000 patient ratings have been collected for back pain, so far. Peleg et al. [6] report that results from this platform correspond to findings of randomized control trials. According to the crowd-sourcing platform itself, postural modifications are the highest-ranked interventions in terms of positive success. Posture training can be supported by wearable devices [7,8] to provide both, the user and the trainer, with real-time feedback about the student’s posture.

In their review of works in the field of wearable technology for spine movement assessment [9], Papi et al. point out that the majority of articles in that field reported on the validity of their system with relatively few works making use of real-world data. They additionally state that the systems used were usually rather cumbersome. While their systematic review focussed on dynamic task performance only including papers themed in this direction, they excluded only three papers from their original compilation of 1610 qualifying papers (1566 of these were excluded based on their title or abstract) because the paper did not focus on *dynamic* task performance. This leads us to the valid assumption that real-world data is generally not widely used in dynamic or non-dynamic movement assessment of the spine.

Methods for analyzing changes in spine shape or posture [10,11,12,13,14] usually need a predefined ‘normal’ state to compare to or prevalently use statistical test to attempt differentiation between two defined groups. Section 2 on related work goes into detail on the aforementioned works.

We aim to address these gaps by systematically analyzing geometric changes in posture as a result of postural training by a Gokhale Method teacher and captured by the *SpineTracker*™ wearable (http://spinetracker.com, accessed 31 July 2019). To this end, we compare snapshots of the measured spine curve before training and the most recent target set by the teacher during or at the end of posture training. Our analysis does not rely on the definition of a global ‘normal’ spine shape.

The main contributions of this paper are:

1. The analysis of postural change in a large real-world data base of posture data, recorded using the SpineTracker wearable, by
  - devising a medium dimensional feature set from the spine curve data, well suited for further analysis and
  - showing how these data can be embedded into a two-dimensional feature space using a combination of standard dimensionality reduction techniques.
2. The demonstration that simple unsupervised clustering in the defined feature space results in a data separation which is geometrically and semantically meaningful.

The novelty of our approach lies in the fact that (a) works making use of real-world data to assess spinal movement, especially with wearables, are very rare, and that (b) we do not rely on any predefined ‘normal’ state to compare to.

Throughout this document we make use of the following terms: we use position to describe a passive, static state, e.g., standing, sitting. The realization of such a state by a person is called posture, emphasizing its execution as a multi-factor dynamic (active) process of both, skeletal alignment and muscle activation. When looking at a temporal sequence of motion data (here posture data), snapshot refers to a single element in that sequence.

The remainder of this work is organized as follows: We give an overview of related works in Section 2. A detailed description of the used materials and methods is given in Section 3. We present the results of our approach in Section 4 and discuss our findings in Section 5. Finally, the paper is concluded in Section 6.

## 2. Related Work

Human motion capturing refers to the recording of human movement and transforming them into a digital 3D representation. Full-body motion capture systems include optical and non-optical systems. Optical systems generally rely on imaging sensors and computer vision algorithms to capture a person with or without a set of passive or active markers attached to their body. Non-optical systems include motion capturing based on e.g., exoskeletons and inertial measurement unit (IMU) based systems. Optical systems generally suffer from being restricted to a capturing volume. Using inertial systems is one way to lift such restrictions.

### 2.1. IMU-Based Full-Body Motion Capture and Reconstruction

Roetenberg et al. [15] describe the Xsens (https://www.xsens.com, accessed 31 July 2019) IMU-based full-body human motion capturing relying on biomechanical models and sensor fusion algorithms. The system uses a set of 17 sensors. Earlier, Tautges et al. [16] enabled full-body animation through four 3D accelerometers attached to wrists and ankles in combination with a large database of motion clips recorded with a marker-based optical motion capture system. Extending on the aforementioned work, Riaz et al. [17] use accelerometer data of wrists and the lower trunk together with ground contact information (computed from trunk sensor) for data-driven motion reconstruction.

### 2.2. Capturing of Body Parts, Specifically the Shape of the Spine

Sometimes it is desirable to only capture data from specific body parts in order to capture these in finer detail. Examples include capturing of the face [18,19], arms [20], legs [21], hands [22,23], and spine [7,10,12,24,25,26,27,28,29,30].

There have been different technologies for measuring the curvature of the spine reaching from image-based surface reconstruction methods (e.g., static and dynamic rasterstereography [24,25], CT scans [10], laser-triangulation [26]) over a ribbon of (eight) fibre-optic sensors, e.g., Williams et al. [27], strips of (twelve) strain-gauge elements, e.g., Consmüller et al. [28], and accelerometers, e.g., Stollenwerk et al. [7], to inertial sensors, e.g., Wong and Wong [12], Cajamarca et al. [29], and Voinea et al. [30].

Wong and Wong [12] developed a smart garment with three inertial sensors (3D accelerometer, three 1D gyroscopes) for posture training. Sensors were mounted between T1 and T2, between T11 and L1, and on S1 (The human spine [31] is divided into four segments (from top to bottom): cervical spine (neck region), thoracic spine (mid-back), lumbar spine (lower back), and sacrum (base of the spine). Each of these regions consists of several vertebrae (here listed from top to bottom). The cervical spine consists of seven vertebrae, abbreviated C1 through C7. The following twelve vertebrae belong to the thoracic spine and are abbreviated T1 through T12. Beneath the thoracic spine are the five lumbar vertebrae, L1–L5. The sacrum is a triangular-shaped bone located below L5. It consists of five fused sacral vertebrae S1–S5). The authors estimated inclination angles in the sagittal and coronal plane of thoracic and lumbar spine and measured posture change as a change in inclination between pairs of neighboring sensors. They also included a small study on the garment’s posture feedback system concluding that it helped participants to avoid poor postures.

Cajamarca et al. [29] built StraightenUp+, a low-cost wearable device for monitoring posture explicitly designed for older persons. StraightenUp+ is a backpack-shaped waist-adjustable harness vest in which three inertial sensors (3D gyroscopes and 3D accelerometers), along with other necessary hardware, are attached to the vertical rear strap. Sensors are distributed equidistantly along the vertical strap covering approximately the full length of the back. They use the sensor data to identify a fixed set of eight physical activities.

Voinea et al. [30] and Stollenwerk et al. [7] describe a 2D reconstruction model for the shape of the human spine based on inertial sensors and plain accelerometers. Although Voinea et al.’s sensors are capable of outputting 3D orientations, they only use a single angle for spine shape reconstruction. In their setup, the five sensors are distributed equidistantly between C7 and L4 vertebrae. While from a reconstruction image their model looks a lot like the one used in [7], Voinea et al.’s models are designed to explicitly represent a C-shaped spine (kyphosis) and an S-shaped spine (normal), the authors of [7] do not make that assumption. Another difference is that [7] puts the first sensor on the L4 vertebra but distributes the following sensors equidistantly over a fixed-length segment independent of the person’s spine length.

An overview of commonly used technologies for spine movement assessment along with respective spine outcomes is reported in the Papi et al. review of works in the field of wearable technology for spine movement assessment [9]. As explained in detail in the introduction (Section 1), this work gave rise to our assumption that in general, real-world data is rarely used in the movement assessment of the spine, neither in dynamic nor in non-dynamic settings.

### 2.3. Methods for Data Analysis

Hay et al. [10] compare spine curves extracted from CT images to a model spine curve computed from multiple individuals without spinal disorders and a history of back pain. Aiming at the detection and quantification of pathologies, the comparison is based on the amount of curve deviation from the model, the deviation of the curvature as well as the torsion along the curve.

In their paper, Brink et al. [11] evaluate the amount of postural change in adolescent computer users after a period of twelve months in order to understand associations between postural change and upper quadrant musculoskeletal pain. For this purpose, different sitting postural angles were recorded and individually analyzed with respect to magnitude and orientational change using univariate and multivariate linear regression models. Sitting postural angles considered were head flexion, neck flexion, craniocervical angle, and trunk flexion. These angles mainly target the upper spinal region. In a small three-day posture feedback study (first day without feedback, second and third day with feedback) Wong and Wong [12] compare average trunk angles between days with and without feedback as well as between days with feedback.

Franklin and Conner-Kerr [13] investigated the relationship of postural changes during pregnancy and back pain. They measured and compared means and standard deviations as well as state analysis of variance (ANOVA) results for a total of nine postural variables (seven postural angles and head and shoulder displacements) of women in the first and third trimesters of pregnancy. Gonzáles-Sanchez et al. [14] compare thoracolumbar curvature angles between two groups (normal weighted and obese persons) using Student’s t-test (parametric test for independent data) and Wilcoxon’s test (non-parametric tests) in order to find out if there are statistically significant differences between the two groups.

As stated before (Section 1), methods summarized here either rely on a predefined ‘normal’ state to compare to or use statistical tests to compare two defined groups. In contrast to this, we aim at an analysis of changes in spinal shape essentially comparing two arbitrary spine curves of one person at two points in time, one before (unguided) and one after posture training (guided). To the best of our knowledge, there is no method for the geometric analysis of that change.

## 3. Materials and Methods

In this section we describe the system used for data recording in Section 3.1, the data we worked with (Section 3.2) as well as the derived feature space (Section 3.3) and dimensionality reduction techniques in Section 3.4 and Section 3.5. The following Section 3.6 contains information on minimum spanning trees and how they can be used for clustering. We detail our processing pipeline in Section 3.7 and conclude with an explanation of visualization methods used to present results (Section 3.8).

### 3.1. Wearable

**Figure 1:**
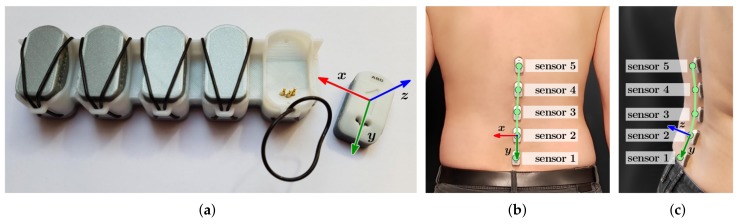
Photos of the *SpineTracker* sensor system. (**a**) Four of the five sensors are sitting in the charger. The sensor outside the charger is shown with its local coordinate system. A single sensor has the dimensions 33mm×16mm×10mm. Each sensor is attached to a person’s lumbar spine with double sided tape. (**b**,**c**) Back and side view of sensor positioning on the lumbar spine including directions of the sensor coordinate system. Sensors are overlayed with a reconstructed spine curve (green dots and line).

The system used for capturing the spinal shape is the *SpineTracker* developed by Gokhale Method Enterprises, Stanford, CA, USA. It consists of five individual accelerometer-based sensors (Figure 1a) which are attached to the lower back of the user as shown in Figure 1b,c. In contrast to single-device posture wearables, the five-sensor approach enables the capture of more detailed spinal curvature information also covering a larger portion of the spine.

All five sensor units were technically identical. The sensors connected and streamed data wirelessly to one host via bluetooth; currently this may be an iOS device or a computer. The sensors support sample rates of up to 50 Hz, thus allowing for a variety of applications, ranging from slow-motion measurements, e.g., sitting, to faster movements such as brisk walking.

More detailed technical information on the wearable as well as the model used for reconstruction of a spine shape from the accelerometer readings and the systematic evaluation of the system’s accuracy (sensors and reconstruction method) can be found in [7].

### 3.2. Data

The data representing the shape of the spine is usually either a set of angles or a set of 2D positions: when streaming data from the sensors, spine shape information is stored as a sequence of forward tilts $\tau_{\mathrm{acci, i}},i=1,\ldots ,5$ of the five *SpineTracker* sensors ordered by sensor ID. Using the spine curve model described in [7], these five angles are transformed into a 2D spine curve consisting of six 2D points $P_{j}$ (a base point $P_{0}$ and five sensor positions $P_{i},i=1,\ldots ,5$) and arc segments connecting these points. For consistent visualization, the spine curve is positioned in space in a way that the first sensor lies in the origin, $P_{1}=\left(0,0\right)$.

The *SpineTracker* sensor system can also capture single frames (“snapshots”) from the data stream that will represent the shape of the spine at that point in time. We used a database of such snapshots of three distinct positions: standing, sitting, and (hip) hinging. Snapshots can be unguided or guided. In an unguided snapshot, a person assumed one of the positions on their own and without a trainer’s intervention or support. A guided snapshot was taken when the position was assumed under the guidance of a posture trainer. Each single snapshot in this database was labelled either “guided” or “unguided”.

From this database, we considered per-user snapshot pairs. These pairs consist of one unguided posture and one teacher-guided posture in which the highest amount of change is to be expected. i.e., for the unguided postures, we extracted the respective initial posture snapshot of a position ($t_{0}$ snapshot). For the guided postures, we used the latest guided snapshot ($t_{1}$ snapshot) available. The *$t_{0}$ snapshots* hence represent the most unlearned realizations of the position and the *$t_{1}$ snapshots* contain the current best imitation of the position the user aims for at the moment of capture. As a consequence, we use exactly one pair per user and position even if more snapshots of that user are available for a specific position. For ethical reasons all data was anonymized. Table 1 gives details on the size of the database used, split by position, as well as overall. It additionally shows statistics on the time passed between unguided and guided snapshots.

In order to show that there was a significant difference in angles between the $t_{0}$ and $t_{1}$ snapshot pairs in one or more sensors, indicating plausibility of further analysis, we considered each sensor individually and conducted a paired samples Wilcoxon test (also called the Wilcoxon signed-rank test) [32].

The database of ($t_{0}$, $t_{1}$) posture pairs used in this work is publicly available at https://skylab.vc.h-brs.de/kstoll2m/PosturePairsDB19/ (Supplementary Materials).

### 3.3. Feature Extraction

For each pair of snapshots in the database of position-posture-pairs we compute a feature vector *F*. *F* is composed of the normalized difference $d_{j}$ of the sensor positions in the $t_{0}$ and $t_{1}$ snapshots and the length $l_{j}$ of the difference

(1) $$\begin{array}{c} l_{j}={∥P_{j|t_{1}}-P_{j|t_{0}}∥}_{2} \end{array}$$

(2) $$\begin{array}{c} d_{j}=\frac{P_{j|t_{1}}-P_{j|t_{0}}}{l_{j}}, \end{array}$$

where $P_{j|t_{0}}$ ($P_{j|t_{1}}$) is the *j*-th 2D spine curve point in snapshot $t_{0}$ ($t_{1}$). This way, spinal shape change is expressed by the 2D directional change between the two snapshots and the 1D amount of change. This results in a 15 dimensional feature vector, five 2D directions $d_{j}$ and five 1D lengths $l_{j}$, j∈{0,2,3,4,5}, for each snapshot pair. We only considered five of the six spine curve points leaving out the sensor positioned in zero ($P_{1}$). For general statistics we continue to use the sensor angle data $\tau_{\mathrm{acci,i|t}}$, i=1,…,5, $t\in \{t_{0},t_{1}\}$.

### 3.4. Principal Component Analysis

Principal component analysis (PCA) [33] is one of the oldest and most widely used linear dimensionality reduction techniques. It is based on the eigendecomposition of the data’s covariance matrix. Its eigenvectors are sorted by their respective eigenvalue forming the new orthogonal coordinate axes (principal component) of the underlying data set. The directions of the new coordinate axes coincide with the directions of maximum variation of the original data points. Geometrically spoken, PCA rotates the Cartesian coordinate system of a high dimensional point set in a way that maximizes the variance of the data along each axis. After the transform, axes are sorted by descending variance. Dimensionality reduction is achieved by retaining only the first *n* principal components.

### 3.5. T-Stochastic Neighbor Embedding

T-stochastic neighbor embedding (t-SNE) [34] is a more recent non-linear dimensionality reduction technique for mapping high-dimensional data to a low-dimensional space (often called a map). It is based on the following:

- A fix data similarity matrix of conditional distances between pairs of data points in the original high-dimensional space. Conditional distances are computed based on a combination of the pairwise Euclidean distances and a point-specific Gaussian distribution.
- A similar point-wise neighborhood estimation in the low-dimensional target space (map similarity matrix), exchanging the Gaussian distribution with a one degree-of-freedom Student’s t-distribution.

The goal is to iteratively adapt the map similarity matrix to best fit the data similarity matrix. This is achieved by minimization of the Kullback–Leibler divergence [35] of the two probability distributions underlying the two similarity matrices.

### 3.6. Minimum Spanning Tree and Clustering

A minimum spanning tree (MST) is a graph theory concept fulfilling the following criteria: given an undirected graph G=(V,E) of vertices *V* and weighted edges *E*, an MST is a tree $\left(V,E^{\prime }\subseteq E\right)$ including all vertices of *G* using a minimum number of edges $E^{\prime }$ such that the total weight of the edges is minimal. The MST is unique if all edge weights are distinct, which is a reasonable assumption for real-world scenarios.

MSTs can be used for clustering. Let G=(V,E) be a fully connected graph of the data points (*V*) of a data set. Let the weight of each edge e={v,w}∈E be defined as e.g., the Euclidean distance between the data points *v* and *w* it connects. This graph is used to construct an MST which is then used for clustering: Subsequent cutting of edges with highest weight increases the number of connected components in this graph. Connected components represent clusters.

### 3.7. Processing Pipeline

**Figure 2:**
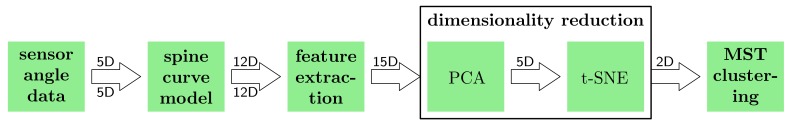
Overview of the processing pipeline used. Individual steps are marked by boxes, arrows indicate the direction of the pipeline. Each arrow is annotated with dimensionality of the data output by the preceding step. Two dimension labels indicate that the data from the two postures in each posture pair is not yet combined.

An overview of our processing pipeline is given in Figure 2. For each pair of snapshots in our database, we compute each posture’s 2D spine curve and extract one feature vector per snapshot pair as described above (Section 3.3). We perform PCA to the set of extracted features to reduce the dimensionality of the data while retaining over 95% of the feature data’s variance. We additionally use t-SNE to further reduce the dimensionality to two dimensions. PCA, as well as other linear dimensionality reduction techniques, preserves the global structure of the data. This preservation of global structure results from maintaining a high variability in the data which in turn translates in a separation of dissimilar data points. The non-linear dimensionality reduction t-SNE tries both, keep similar data points close together and dissimilar data points far apart. This is particularly interesting for finding clusters of data points in higher dimensional space.

Clustering generally groups data points sharing a set of properties. Even though postural training is a highly individual process, we assume that the recorded data will exhibit certain differences and commonalities. This in turn will allow us to combine posture pairs into distinct clusters. We apply MST-clustering to the two-dimensional t-SNE map of the chosen feature space, as there is no prior knowledge on the general structure of clusters in the 2D t-SNE map. We compute the Euclidean distance between all pairs of t-SNE map points, which results in a similarity matrix of the map. The MST is constructed on this similarity matrix. Using MST-clustering is a reasonable choice due to the following properties:

1. The t-SNE map equalizes the density of neighboring data points in the higher dimensionality evening out distances between neighboring points in the map. As a consequence condensed clusters are spread and spread clusters are contracted.
2. In MST-clustering, data points are grouped together by proximity and need neither be separable by a regular geometric curve nor grouped around a centroid.

### 3.8. Visualization of Results

**Figure 3:**
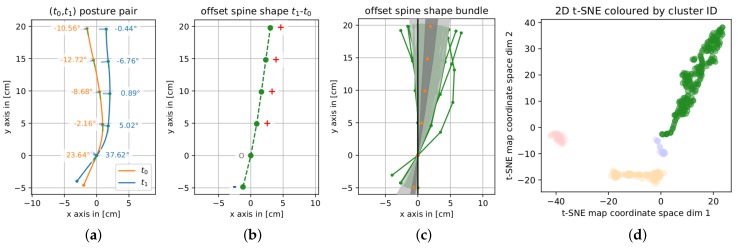
(**a**) Two spine shapes reconstructed from the $t_{0}$ and $t_{1}$ input angles and (**b**) resulting *offset spine shape*. (**c**) Offset spine shape bundle representing a data cluster in the feature set’s T-stochastic neighbor embedding (t-SNE) map (**d**), colored by cluster ID.

We visualize clustering results using the 2D t-SNE map of the data coloring each point by its cluster ID. We compute the angle difference between the $t_{1}$ and $t_{0}$ snapshots, $\tau_{{\mathrm{acci,i|t}}_{1}}-\tau_{{\mathrm{acci,i|t}}_{0}}$, i=1,…,5. This set of difference angles was used to reconstruct an *offset spine shape*. The resulting offset base position $P_{\mathrm{offset},0}$ and offset sensor positions $P_{\mathrm{offset},i},i=1,\ldots ,5$, ordered by increasing *y*-coordinate, represent the computed differences as offset from a vertical line. Figure 3b shows an example of such an *offset spine shape*. The underlying two reconstructed spine shapes are illustrated in Figure 3a.

Spine shapes are annotated with the sensor angle data they were constructed from and an indication to which side the person is looking: the line in each sensor position points away from the person’s back.

*Offset spine shapes* are annotated with the sign of each offset position’s *x*-coordinate. A red ‘+’ stands for positive, a grey ‘o’ for zero, and a blue ‘−’ for a negative horizontal offset from a vertical line positioned in zero.

As each data point in a cluster represents the difference of two spine shapes, we also visualize clusters by overlaying all offset spine shapes of each cluster as displayed in Figure 3c. The light grey area in this visualization represents the region between the 1st-percentile and 99th-percentile of the horizontal distribution of the data. Covering the central 50% of the horizontal extent of the offset spine shapes (at each point level), the dark grey area shows the region between the 25th-percentile and the 75th-percentile. Orange dots mark the median of the horizontal extent of the data. This overlay serves as visualization of the main orientation of the offset spine shapes in a specific cluster.

## 4. Results

In the following, we first list the results of the angle data analysis (Section 4.1) and motivate why this analysis is relevant. We then describe the behavior of the clustering under variation of the t-SNE parameter perplexity in Section 4.2. For this section, too, we state reasons for analyzing results from this variation. The last Section 4.3 presents and analyzes the results of the clustering of the posture data divided by position based on their geometry.

### 4.1. Analysis of the Angle Data

As normality test (Shapiro-Wilk [36], Anderson-Darling [37], Q-Q Plot [38]) indicated that the data is not normally distributed, we use the non-parametric paired samples Wilcoxon test (also called the Wilcoxon signed-rank test) [39] to check whether there was a difference in posture for the unguided and guided snapshots already on a single sensor level. We could confirm that for all positions, there was at least one sensor in which the median change in angle between the unguided $t_{0}$ snapshots and the guided $t_{1}$ snapshot pairs was significantly different from zero.

For standing, there was a significant difference in angle between the $t_{0}$ and $t_{1}$ snapshot pairs for all sensors ($p<\varepsilon =10^{-6}$). For sitting the difference between the two snapshots is significant for only the first sensor (p<ε). Finally, the angle difference between $t_{0}$ and $t_{1}$ snapshot pairs is below the standard significance threshold α=0.05 for all but one sensor for hip hinging (p<ε for four out of five sensors).

**Figure 4:**
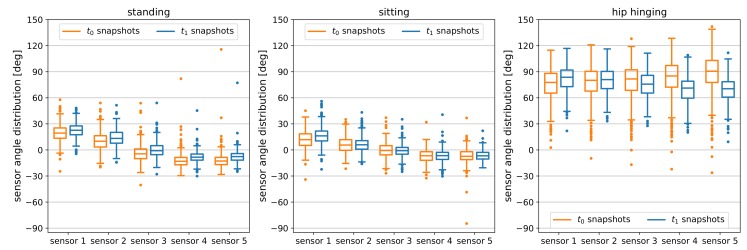
Boxplots of the angle distribution of the $t_{0}$ and $t_{1}$ snapshots grouped by sensor. The box frames the lower ($Q_{1}$) to upper ($Q_{3}$) quartile values of the data. The horizontal line inside each box marks the data’s median. Whiskers include data between $Q_{1}-1.5\;\mathrm{IQR}$ and $Q_{3}+1.5\;\mathrm{IQR}$, where $\mathrm{IQR}=Q_{3}-Q_{1}$ abbreviates the interquartile range. Outliers outside the whisker range are marked with dots.

Descriptive statistics of both snapshots for all positions and sensors are listed in Table 2. The last column of the table marks the previously reported sensors for which p<α. Figure 4 provides additional information on the distribution of the five sensors’ angle data for each position.

### 4.2. Clustering under Varying t-SNE Perplexity

**Figure 5:**
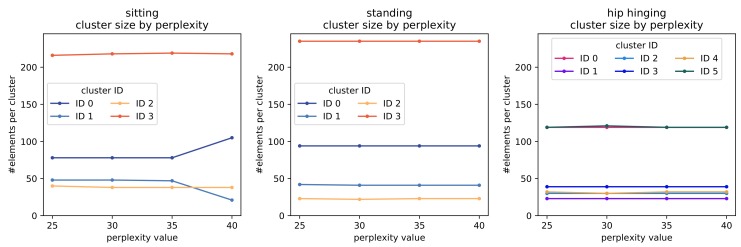
The number of elements per cluster under variation of the perplexity value, plotted by cluster ID.

The t-SNE parameter *perplexity* reflects the number of neighbors expected in its distance optimization process. In their paper [34] van der Maaten and Hinton state that variation of perplexity has little influence on the performance of t-SNE. In order to assert clustering of our data is prevalently consistent under variation of perplexity, we show the results of clustering the 2D t-SNE map into four (six for hip hinging) clusters for perplexity values ranging from 25 to 40 in steps of five in Figure 5.

**Figure 6:**
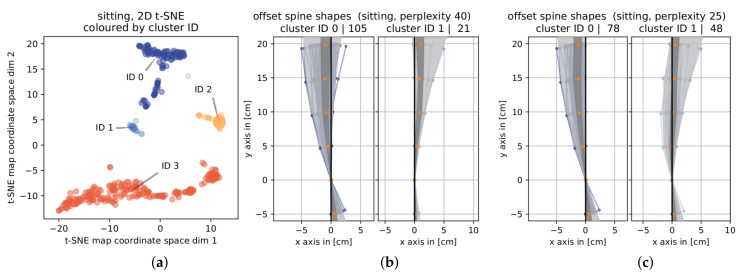
Visual comparison of changes in clustering when varying perplexity. (**a**) A 2D t-SNE map and (**b**) offset spine shape bundles per cluster for a perplexity value of 40. (**c**) offset spine shape bundles per cluster for a perplexity value of 25. The offset spine shape bundles displayed represent the only two clusters that changed for a perplexity value of 40. All axis titles of the offset spine shape bundles contain information of the cluster ID and the number of elements in that cluster.

As Figure 5 shows, most of the time the general size of the clusters were approximately identical under variation of perplexity. This was also true for the data represented by these clusters. The only noteworthy exception is observed in *sitting* when using a perplexity value of 40, also see Figure 6a,b in comparison to Figure 6c, which is drawn using a perplexity value of 25 but is the same for 30 and 35. While the general shape of the t-SNE map superficially appears unchanged, distances between groups of data points slightly shift. The three segments of cluster 1 were pulled apart and partially moved closer to cluster 0, thus changing the split position of clusters 0 and 1 and associating about 25 data points more (less) to cluster ID 0 (1) than for lower perplexity values.

Inspecting the data (offset spine shape bundles) more closely, we see that with a perplexity value of 40, almost all offset spine shapes in cluster ID 1 havd a positive *x*-coordinate, while this was not the case for lower perplexity values. For lower values, these two clusters are predominantly split based on the horizontal offset from zero in the base position $P_{0}$ and the topmost position $P_{5}$. The data in the other clusters remain identical throughout parameter variation.

#### Choice of Parameters for Cluster Analysis

Throughout the results presented in this paper we used the following parameters. The number of PCA components was chosen such that the resulting lower dimensional space still captures over 95% of the feature data variance. For the computation of the 2D t-SNE map, we fixed the cost parameter perplexity to 30, which is approximately the centre of achieving repeatedly stable results for clustering (see Figure 5). Optimization parameters of t-SNE are left to the implementation defaults [40] as these in general only affect the rate of convergence [41]. Upon initial inspection of the data, and after confirming that clusters would be stable when varying parameters in repeated computations, we set the number of clusters to four for standing and sitting and to six for hip hinging.

### 4.3. Analysis of Clusters per Position

The following three sections analyze results from clustering from a geometric point of view, assigning a geometric meaning to each cluster. The analysis is separated into the three positions: standing Section 4.3.1, sitting Section 4.3.2, and hip hinging Section 4.3.3.

#### 4.3.1. Standing

**Figure 7:**
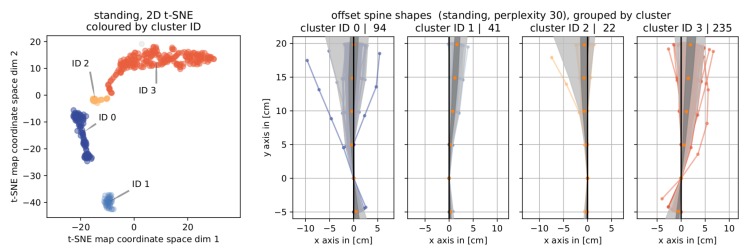
Results for clustering posture pairs of *standing*. (**left**) The T-SNE map labelled with and colored by cluster ID. (**right**) Corresponding cluster bundles of *offset spine shapes* including information about the cluster ID and the number of elements in that cluster in each axis title.

**Figure A2:**
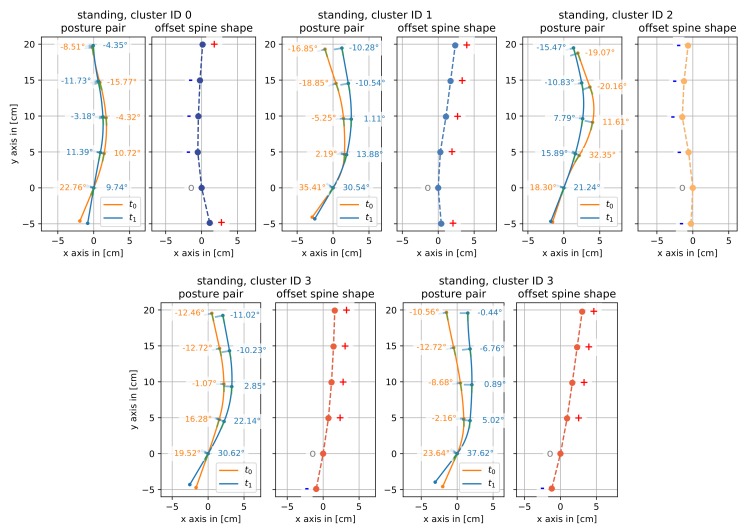
Results for clustering posture pairs of *sitting*: Cluster representative sample posture pairs and offset spine shapes for each cluster. Each sample’s position within its cluster is marked by an ‘x’ in the colored t-SNE map.

Figure 7 illustrates the results of clustering the data from posture pairs of *standing* into four components in form of their 2D t-SNE map and in form of offset spine shape bundles. For the visualization of cluster representatives ($t_{0}$ and $t_{1}$ posture pairs and $t_{1}-t_{0}$ offset spine shapes), please see Figure A2 in the Appendix A.

Geometrically, the four clusters were separated mainly based on the position of the offset spine shapes’ base points and further by the orientation of the upper part. For the first and second cluster (IDs 0 and 1), the offset spine shapes all had positive base point *x*-coordinates while these were negative for the third and fourth cluster (IDs 2 and 3). The first and second cluster differ in the position representing the second sensor ($P_{\mathrm{offset},2}$), for ID 0 its *x*-coordinate is always negative while it was always positive for ID 1). The upper part of the offset spine shape bundle of cluster ID 0 shared no particular orientation, this part is clearly leaning in positive *x* direction. This pattern was repeated for the third and fourth cluster (IDs 2 and 3), in which the third cluster tends to negative values and the fourth cluster leans to positive values. Again a clear geometric separation can only be seen for the offsets in $P_{\mathrm{offset},2}$.

#### 4.3.2. Sitting

**Figure 8:**
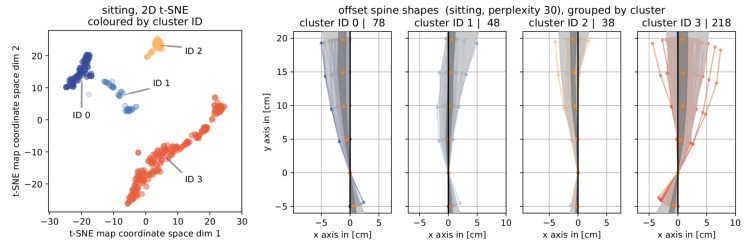
Results for clustering posture pairs of *sitting*. (**left**) The t-SNE map labelled with and colored by cluster ID. (**right**) Corresponding cluster bundles of *offset spine shapes* including information about the cluster ID and the number of elements in that cluster.

**Figure A4:**
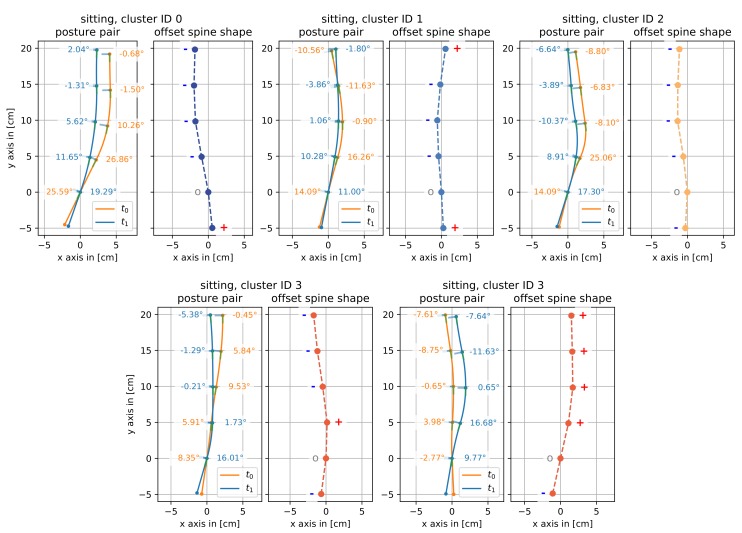
Results for clustering posture pairs of *sitting*: Cluster representative sample posture pairs and offset spine shapes for each cluster. Each sample’s position within its cluster is marked by an ‘x’ in the colored t-SNE map.

Figure 8 shows the results of clustering the data from sitting posture pairs into four components using the 2D t-SNE map and the offset spine shape bundles per cluster. Again, we show representative spine curve shapes and offset spine curve shapes for each cluster in the Appendix A, Figure A4.

The geometric pattern observed in *standing* repeats for *sitting*: the four clusters are separated mainly based on the position of the offset spine shapes’ base points and further by the orientation of the upper part: For the first and second cluster (IDs 0 and 1), the offset spine shapes all have positive base point *x*-coordinates while these are negative for the third and fourth cluster (IDs 2 and 3). The two groups can each be further separated into positive slanted clusters (IDs 1 and 3) and negative slanted clusters (IDs 0 and 2). While only sensor 1 shows a significant difference between the angular data of the unguided and guided snapshot pairs on a per sensor level (see Section 4.1 and Table 2), the combined information on all five sensors allows for a class separation into four clusters.

#### 4.3.3. Hip Hinging

**Figure 9:**
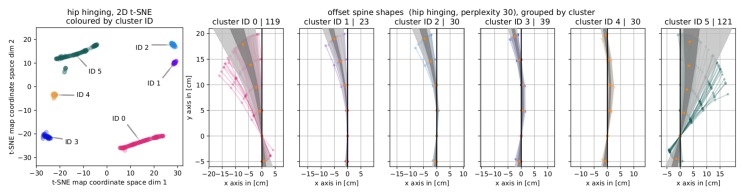
Results for clustering posture pairs of *hip hinging*. (**left**) The t-SNE map labelled with and colored by cluster ID. (**right**) Corresponding cluster bundles of *offset spine shapes*.

**Figure A6:**
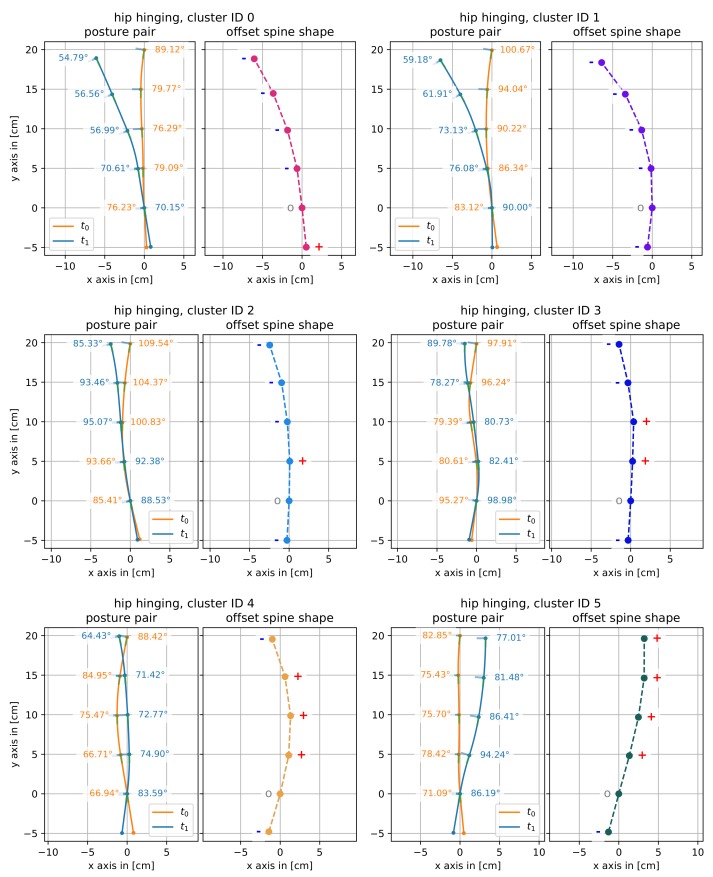
Results for clustering posture pairs of *hip hinging*. Cluster representative sample posture pairs and offset spine shapes for each cluster. Each sample’s position within its cluster is marked by an ‘x’ in the colored t-SNE map. The two posture snapshots are additionally rotated about the origin such that the topmost sensor of the unguided $t_{0}$ snapshot lies on the *y*-axis.

The results of clustering $t_{0}$ and $t_{1}$ posture pairs of the position hinging into six clusters are presented in Figure 9. As before, we show the 2D t-SNE map colored by cluster ID along with the corresponding offset spine shape bundles. For representative spine curves for each cluster, please refer to the Appendix A, Figure A6.

Here, the geometry of the offset spine shapes reveals a clear pattern. Again, an initial distinction between the clusters can be made based on the position of the base points. One cluster (ID 0) had only positive *x*-valued base points while this is purely negative for four of the five others (IDs 1–4) and almost purely negative (except for seven samples) for the last cluster (ID 5). In the upper part, all samples in the first cluster (ID 0) more or less strongly slanted to the left, all upper offset spine shape points having a negative *x*-coordinate. This was also the case for the upper part of the second cluster (ID 1). For the subsequent clusters (IDs 2–5) the position at which the offset spine shapes of that cluster cross the thought vertical line passing through x=0 moved stepwise up. For cluster ID 1 there was no crossing. In cluster ID 1, the offset spine shapes cross that line between $P_{\mathrm{offset},2}$ and $P_{\mathrm{offset},3}$, i.e., the *x*-coordinate of all $P_{\mathrm{offset},2}$ is >0 and it is <0 for all $P_{\mathrm{offset},k},k\in \left\{3,4,5\right\}$. For the next cluster (ID 2), the crossing is between $P_{\mathrm{offset},3}$ and $P_{\mathrm{offset},4}$. This progression continues up to the last cluster (ID 5) in which there is no crossing in the observed area, meaning that all offset spine curves slant in positive direction (x>0 for $P_{\mathrm{offset},k},k\in \left\{2,3,4,5\right\}$). These properties defines the separation of clusters with IDs 1–5.

## 5. Discussion

This section shortly summarizes results (Section 5.1 and Section 5.2), puts this work in context with existing research (Section 5.3), and lists limitations in Section 5.4.

### 5.1. Summary of Results

For all three positions, sitting, standing, hip hinging, we found a significant change in posture between the sets of guided and unguided snapshot pairs. We used a Wilcoxon signed-rank test on the sensor angle data representation of the posture pairs. This results indicated that is is plausible to further analyze posture pairs in our data base with respect to postural change.

We confirmed that our clusters remain stable under variation of the t-SNE parameter perplexity.

In the geometric analysis of the clusters for each position, it turned out that clusters formed based on the position of offset spine shapes, the base points, and the direction of the upper part. This underlines the necessity of a multi-sensor system to achieve a meaningful separation into different clusters. The separation of the upper part was more fine-grained as there are six clusters for hip hinging.

Showing several samples per cluster to a professional posture trainer, the samples and clusters, that are based on our geometric feature, had a meaning for her. Her assessments are summarized in the following section.

### 5.2. Sample-Based Evaluation per Cluster by a Professional Posture Trainer

Several samples of unguided and guided posture pairs per cluster were shown to a professional posture teacher. She has experience in comparing such posture pairs and is able to understand how the change in posture within such a posture pair affects the shape of the spine as well as posture in general. This information is important to give semantic meaning to our clusters which formed based on geometric features.

A professional posture trainer interprets clusters for *standing* (see Figure 7) the following way: Cluster ID 0 represents a loss of pelvic anteversion and often (but not always) a reduction in sway. The second cluster (ID 1) stands for a group of users who have sacrificed a little in pelvic anteversion and very much straightened out a sway. The most notable property of the third cluster (ID 2) was a high reduction of sway. The second property which this cluster shares with the last cluster but to a smaller degree is an increased pelvic anteversion. The last cluster cluster could not be attributed with a definitive property describing the changes in the upper part.

From the perspective of a professional posture trainer, in *sitting* (see Figure 8), cluster ID 0 represents cases that have less curvature in the upper lumbar area and (possibly due to having reduced that curvature) reduced pelvic anteversion. The second cluster (ID 1) stands for sacrificing (a little) pelvic anteversion and straightening out a sway. Both remaining clusters (IDs 2 and 3) exhibit an increase in pelvic anteversion. Only cluster ID 2 could clearly be associated with a reduced sway while for the fourth cluster (ID 3) there was no clear attribution to a consistent change in posture valid throughout the cluster.

The posture trainer’s assessments of the clustering for *hip hinging* (see Figure 9) was this: Cluster ID 0 in the guided $t_{1}$ snapshots did not go as far with the pelvis as in the unguided $t_{0}$ snapshot and learned to stop rounding the upper part of the back in order to reach deeper. People in cluster ID 1 go further down in $t_{1}$ than in $t_{0}$ or as deep as in $t_{0}$ in the lowest part of the pelvis and also stopped rounding the upper back. The next cluster (ID 2) represents students who stopped rounding in the upper lumbar area and who have increased their pelvic anteversion. Cluster four (ID 3) is a group with more anteversion in the pelvis and a straighter upper back. This general pattern repeats for cluster ID 4: rounding in $t_{0}$, not rounded any more in the $t_{1}$ snapshot and slightly more pelvic anteversion. In the last cluster (ID 5) the posture trainer sees students who use a sway to overcome their inability to tip their pelvis (alone), thus their pelvic tip increases but so does their sway.

### 5.3. Relation to Existing Research

Previous work on spine shape analysis has for a long time been majorly interesting in medicine. There, analysis is mainly driven by pathology quantification, by relation investigation, or by evaluation of a new technology. Pathology quantification (e.g., Hay et al. [10], 24 participants) is often tightly tied to a definition of a `normal’ spine shape, determining and assessing a given spine shape by its deviation from the ‘normal’ one. Relation driven investigations give answer to questions like *is there a relation between weight groups (normal, obese) and differences in spinal curvatures?* ([14], 39 participants), or *is there a relation of back pain and changes in spine curvature during pregnancy?* ([13], twelve participants), or *is upper back pain related to the amount of postural change in young computer users?* ([11], 153 participants). Relation driven questions are tested for significance and hypothesis confirmation using statistical tests, e.g., ANOVA, Student’s t-test, or Wilcoxon’s test.

Wearables have become an increasingly promising tool for posture monitoring and analysis. Posture change comparisons here are also often based on a ‘neutral’ or ‘normal ’ position: In an evaluation study, Wong and Wong [42] (three participants, three repetitions of each position) use accelerometers to measure postural change in terms of curvature variation from a *neutral sitting* position for three pre-defined modifications: sitting with left (right) lateral bending, and sitting while flexing forward. They computed curvature variation with respect to *neutral sitting*.

Without the notion of a ‘normal’ state for spine shape or posture and without pre-defined groups to put into relation to one another, we invert the process described above: Based on a database of spine shapes of unguided and guided posture pairs, we cluster changes in spinal shape. Clusters form based on the geometry of the change and represent a semantically meaningful separation of the data according to the assessment of a professional posture trainer.

### 5.4. Limitations

Our database did not have information on gender, age, height, or weight of the users. Therefore, we could not separate our analysis based on such criteria. However, these factors can have a considerable impact on the shape of the spine: Nachemson et al. [43] reported a minor tendency for the influence of age on increased stiffness of intervertebral discs. The authors also found that in bending, female motion segments are more flexible than male. According to Hay et al. [10], the BMI, which is based on weight and height, is related to thoracic sagittal kyophosis. Youdas et al. [44] found an association between BMI and pelvic inclination.

Another limitation of our presented work is the choice of the number of clusters. These were mainly based on observations in the 2D t-SNE map. Especially in light of the posture trainer’s evaluation of the clusters, it is easily seen that the number of clusters suggested by the geometry does not necessarily reflect the number of clusters found by the posture trainer. Therefore it would be highly beneficial to include several posture trainer’s knowledge on patterns in change into the clustering approach and maybe even into feature design.

We showed several samples per cluster to only one professional posture trainer for postural change evaluation. As this work aimed at describing a general methodology for analysis of changes in spine shape, we did not do a blind-test cross-validation with several posture trainers, removing any information relating a sample to a cluster and have several posture trainers assess the samples. However, this would make the evaluation of the postural change in the clusters independent and improve the reliability of the clusters that formed based on geometric features and the trainers’ assessments.

## 6. Conclusions and Future Work

This work aimed at analyzing the change of the spinal shape under posture training in three different positions: sitting, standing, and hip hinging. In particular, it compared snapshots of an unguided-guided posture pair based on features computed from the 2D spine curve geometry. Clustering was used to group posture changes with common geometric characteristics. The results from clustering our spine-shape-geometry inspired features could be identified with specific changes in posture by a professional posture trainer. Our large data base consists of real-world spine curves of over 350 single-user posture pairs and is pathology-unrelated. Our analysis is independent from a definition of a global *normal* spine shape. Instead, it is a highly individual process based on each individual’s spine shape before and after posture training. This makes the successful separation of change in spinal shape into geometrically and semantically meaningful clusters (second contribution stated in the introduction, see Section 1) both interesting and important. We believe that this is the first work in the field of wearable-sensor-based evaluation of spine curves that analyzes this many distinct data points based on geometric features without relying on the definition of a *normal* spine shape.

Future work includes blind-test cross-validation of multiple sample posture pairs per clusters by several posture trainers as well as a more detailed grouping of our data based on additional information about e.g., age and gender. Further research could also include comparison against pain ratings using standard questionnaires, e.g., the low back pain specific Roland Morris questionnaire, or targeting general health status measurement SF12 and SF36, and eventually associate pain levels or changes therein with certain groups of posture change.

Furthermore it will be interesting to integrate feedback based on common features in spinal shape change, represented by clusters, into posture training. This could e.g., be implemented by also categorising posture training exercises based on a student’s progress and suggest exercises tailored to their group of shape change. Moving partially away from clustering postural change towards analysis of spinal shape change of an individual user could provide them with helpful instant feedback on which region they need improvement on and how to get there. This also could be realized using e.g., an exercise specifically designed to improve the identified region. It would need a previously taken guided snapshot to compare the current user snapshot to.

## Figures and Tables

**Table 1:** General statistics of the data basis used for our analysis. The column labelled # samples contains information on the number of snapshot pairs per position. The # users states how many different users recorded such pairs. The last four columns state the minimum, average, median, and maximum time passed between the snapshots in each pair. Time is displayed as a tuple of days (d), hours (hh), minutes (mm), and seconds (ss).

	# Samples	# Users	Time Difference, Format: “d hh:mm:ss”			
			**Minimum**	**Mean**	**Median**	**Maximum**
standing	393	393	0 00:30:07	30 01:34:18	2 00:39:43	443 03:38:05
sitting	386	386	0 00:30:13	23 23:03:02	2 01:16:43	399 00:23:05
hip hinging	362	362	0 00:37:44	24 11:23:42	2 00:54:51	385 21:39:54
full	1141	425	0 00:30:07	26 05:26:02	2 01:08:07	443 03:38:05

**Table 2:** Mean, standard deviation (sd), minimum (min), maximum (max), and median (med) values as well as the interquartile range (IQR) of all five sensor’s angle data for $t_{0}$ and $t_{1}$ snapshots. A star in the last column stands for a rejected null hypothesis of the Wilcoxon signed-rank test (p< 0.05).

	$t_{0}$ Snapshots						$t_{1}$ Snapshots				
	**Mean (SD)**	**Min**	**Max**	**Med**	**IQR**		**Mean (SD)**	**Min**	**Max**	**Med**	**IQR**	
**standing**												
sensor 1	19.6 (10.3)	−24.6	57.8	19.4	11.8		22.5 ( 8.0)	−4.5	48.2	22.7	10.2	*
sensor 2	10.1 (10.7)	−19.8	54.1	10.0	13.0		13.8 ( 9.2)	−14.4	51.4	13.3	12.4	*
sensor 3	−4.1 (10.3)	−40.4	53.8	−4.5	11.1		−0.1 ( 9.0)	−27.9	54.1	−0.9	10.4	*
sensor 4	−12.3 ( 8.9)	−29.6	82.0	−13.1	8.5		−8.2 ( 6.6)	−30.1	45.3	−8.3	7.0	*
sensor 5	−12.0 ( 9.5)	−28.2	115.7	−12.8	8.3		−7.6 ( 7.3)	−24.9	77.2	−7.7	7.5	*
**sitting**												
sensor 1	12.0 (10.0)	−34.1	45.2	11.8	13.4		16.7 (10.2)	−22.3	56.1	16.2	11.4	*
sensor 2	6.0 ( 9.4)	−21.6	35.1	5.6	12.3		6.3 ( 8.5)	−16.0	43.2	5.9	9.8	
sensor 3	−0.6 ( 8.7)	−26.9	37.1	−0.7	10.6		−1.0 ( 7.1)	−25.1	35.3	−0.9	7.8	
sensor 4	−6.8 ( 7.4)	−32.5	32.0	−6.7	9.9		−6.9 ( 6.9)	−30.3	40.6	−6.6	8.2	
sensor 5	−6.9 ( 8.2)	−84.7	36.8	−7.3	9.1		−6.7 ( 5.9)	−20.5	22.0	−6.8	7.7	
**hip hinging**												
sensor 1	75.5 (18.1)	2.7	114.8	77.7	22.7		81.8 (14.5)	21.9	116.9	83.7	19.1	*
sensor 2	77.4 (18.8)	−9.7	121.0	80.1	22.8		79.9 (14.5)	33.1	116.3	81.0	19.8	
sensor 3	79.1 (20.1)	−16.9	128.1	81.7	23.6		75.1 (15.1)	27.7	111.4	75.7	20.4	*
sensor 4	82.1 (21.6)	−22.2	128.5	85.2	24.9		69.3 (15.1)	20.2	109.1	71.2	19.5	*
sensor 5	87.8 (22.6)	−26.3	142.0	90.7	25.3		69.1 (14.6)	9.4	111.8	70.5	17.8	*

**Figure A1:**
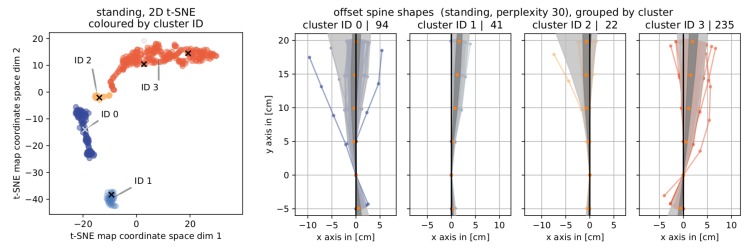
Results for clustering posture pairs of *standing*. (**left**) The t-SNE map labelled with and colored by cluster ID. An ‘x’ marks the position of a cluster representative. (**right**) Corresponding cluster bundles of *offset spine shapes*.

**Figure A3:**
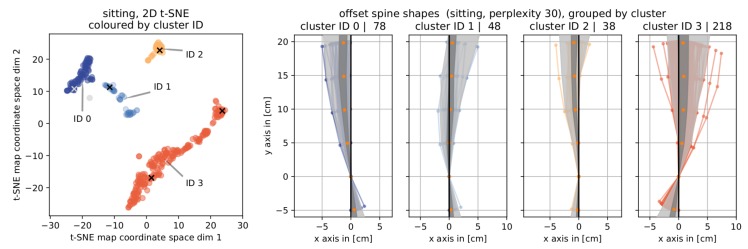
Results for clustering posture pairs of *sitting*. (**left**) The t-SNE map colored by cluster ID. An ‘x’ marks the position of a cluster representative. (**right**) Corresponding cluster bundles of *offset spine shapes*.

**Figure A5:**
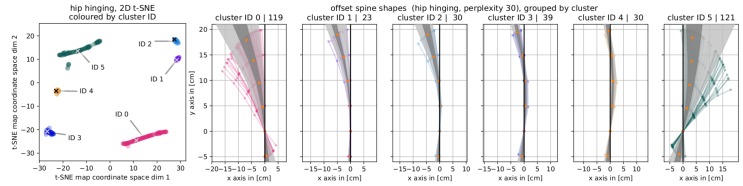
Results for clustering posture pairs of *hip hinging*. (**left**) The t-SNE map colored by cluster ID. An ‘x’ marks the position of a cluster representative. (**right**) Corresponding cluster bundles of *offset spine shapes*.

## Supplementary Materials

The following are available online at https://skylab.vc.h-brs.de/kstoll2m/PosturePairsDB19/: Database of ($t_{0}$, $t_{1}$) posture pairs used in this work.

## Appendix A. Sample Spine Curve of Posture Pairs per Cluster

On the following pages, we will show representative spine curve posture pairs for each cluster. For better overview and localization of presented sample (cluster representative), we re-plot the t-SNE map and corresponding cluster bundles from the results. Each cluster in the t-SNE maps is additionally annotated with the location of the cluster representatives. Figure A1 and Figure A2 draw the results of clustering and sample posture pairs for *standing*. Clusters and representatives for the position *sitting* are displayed in Figure A3 and Figure A4. Finally, Figure A5 and Figure A6 depict clusters and cluster representatives for *hip hinging*.
